# Surgical management of spinal metastases: A cross-continental study in the United States and the Netherlands

**DOI:** 10.1016/j.jbo.2025.100676

**Published:** 2025-03-25

**Authors:** Jantijn J.G.J. Amelink, Bram T. van Munster, Bas J.J. Bindels, Robertus J.B. Pierik, Jasper van Tiel, Olivier Q. Groot, Nicolien Kasperts, Daniel G. Tobert, Jorrit-Jan Verlaan

**Affiliations:** a Department of Orthopaedic Surgery, Division of Surgical Specialties, University Medical Center Utrecht, Heidelberglaan 100, 3584 CX Utrecht, the Netherlands; b Department of Orthopaedic Surgery, Massachusetts General Hospital - Harvard Medical School, 55 Fruit Street, Boston, MA 02114, USA; c Department of Radiation Oncology, Division of Imaging & Oncology, University Medical Center Utrecht, Heidelberglaan 100, 3584 CX Utrecht, the Netherlands

**Keywords:** Spinal metastases, Practice variation, Surgical management, Interdisciplinary therapeutic approaches, Postoperative outcomes

## Abstract

**Background:**

There is currently no consensus on the optimal surgical treatment for patients with spinal metastases. Investigating regional variations in surgical management could provide valuable insights to optimize care and refine surgical practices globally.

**Objective:**

To investigate differences in patient populations, surgical management, and perioperative outcomes among patients who underwent surgery for spinal metastases in either Boston (United States) or Utrecht (Netherlands).

**Methods:**

727 patients surgically treated for spinal metastases in Boston (n=539) and Utrecht (n=188) between 2018–2022 were included. Data on patient characteristics, surgical management, perioperative outcomes, and survival were collected. The Mann-Whitney *U* test was used for continuous data, and Fisher’s exact test for categorical data.

**Results:**

In Boston, a higher percentage of patients had three or more spinal metastases (68% *vs* 59%; p=0.006) and brain metastases (16% *vs* 4.3%; p<0.001) at the time of surgery compared with Utrecht. Vertebrectomy/corpectomy with stabilization was performed in 54% of patients in Boston versus none in Utrecht (p<0.001), whereas percutaneous stabilization was performed in 1.3% of patients in Boston versus 39% in Utrecht (p<0.001). In Utrecht, patients received postoperative radiotherapy more frequently (70% *vs* 54%; p<0.001) and earlier (17 days [IQR:12–24] *vs* 29 days [IQR:23–39]; p<0.001). Postoperative neurological outcome, complications, reoperation rates, and survival did not differ between the two cohorts (all p>0.05).

**Conclusions:**

Differences in patient populations and surgical management exist between tertiary hospitals on separate continents. Further research is needed to determine the optimal extent and timing of surgery to improve quality of life for patients with spinal metastases.

## Highlights

• Differences in patients selected for surgery and surgical management of spinal metastases exist between major cancer centers in the United States and the Netherlands.
• Patients in Boston generally presented with a more advanced stage of metastatic disease at the time of surgery compared with patients in Utrecht.
• Surgical management in Boston typically involved more extensive open procedures, whereas Utrecht favored less invasive percutaneous procedures.
• Future research should determine the optimal extent and timing of surgery to improve quality of life for patients with spinal metastases.

## 1 Introduction

Due to the spine's highly vascular nature, it is the most common site for bone metastases, affecting approximately 20 % of patients diagnosed with cancer [1], [2]. Timely diagnosis and treatment of symptomatic spinal metastases are of utmost importance because of their potential debilitating consequences, such as severe pain, neurological deficit, and autonomic dysfunction [3]. For some patients with symptomatic spinal metastases, surgical intervention may be warranted, aiming to restore spine stability, alleviate epidural compression, and/or perform cytoreduction for local tumor control, which can also be achieved with radiotherapy as in separation surgery [4], [5], [6].

Due to a lack of consensus regarding the ideal surgical management and optimal indication, a wide variety of procedures may be performed for patients with spinal metastases, ranging from minimally invasive percutaneous stabilization to en bloc vertebrectomies/corpectomies [7], [8]. Such variations in surgical management may be attributed to cultural differences, local policies, and diverging philosophies between countries’ healthcare systems regarding best practices, with some prioritizing quality-of-life over quantity-of-life, and vice versa. Additionally, differences in medical characteristics of patient populations (e.g. comorbidities, extent of metastatic disease, amount of prior treatment) may also offer an explanation. Gaining better insight into regional variations in surgical management is crucial. Such understanding allows for the improvement of care worldwide, as surgical research from institutions in the United States and other countries (e.g. the Netherlands, Germany, Switzerland, Japan etc.) is frequently used to inform patient care globally [9], [10], [11], [12], [13].

Therefore, the purpose of the present study is to investigate whether differences exist in patient populations, surgical management, and perioperative outcomes among a large cohort of patients who underwent surgery for spinal metastases at major cancer centers in either Boston (United States) or Utrecht (Netherlands).

## 2 Materials and methods

### 2.1 Study design and setting

This retrospective cross-continental cohort study evaluated data from two affiliated academic hospitals in Boston, the United States and one academic hospital in Utrecht, the Netherlands. The institutional review board at Mass General Brigham in Boston (registration: 2017P00838) approved a waiver of informed consent for this cohort study. The institutional review board at University Medical Center Utrecht (UMCU), under registration VOBU 23U-0528, granted a waiver of additional informed consent to use data from three existing registries of patients who had previously provided informed consent for the prospective collection of baseline demographics, treatment characteristics, and clinical follow-up data [14]. The Strengthening the Reporting of Observational Studies in Epidemiology (STROBE) checklist for cohort studies was used to warrant adequate reporting (Appendix A) [15].

### 2.2 Patient inclusion

Potentially eligible patients for the Boston cohort were identified using the International Classification of Diseases, Tenth Revision (ICD-10) diagnosis of secondary malignant neoplasm of bone and bone marrow (C79.5), diagnosis of multiple myeloma (C90.0), along with all CPT codes related to surgical procedures on the spine and vertebral column. For the Utrecht cohort, potentially eligible patients were identified from three registries: the Global Spine Tumour Study Group (GSTSG), the Metastatic Tumor Research and Outcomes Network (MTRON), and the PRospective Evaluation of interventional StudiEs on BoNe meTastases (PRESENT) cohort, all of which include patients undergoing surgery and/or radiotherapy for metastatic bone disease at the UMCU [16].

**Fig. 1:**
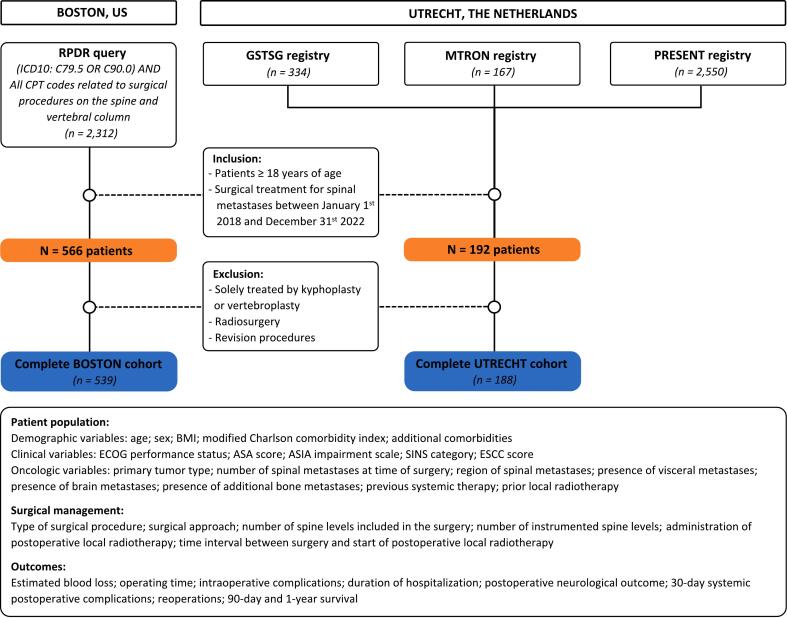
Flowchart of inclusion and exclusion of patients in the study. US = United States; RPDR = Research Patient Data Registry; GSTSG = Global Spine Tumour Study Group; MTRON = Metastatic Tumor Research and Outcomes Network; PRESENT = PRospective Evaluation of interventional StudiEs on BoNe meTastases; N = number; BMI = Body Mass Index; ECOG = Eastern Cooperative Oncology Group; ASA = American Society of Anaesthesiologists; ASIA = American Spinal Injury Association; SINS = Spinal Instability Neoplastic Score; ESCC = Epidural Spinal Cord Compression.

We included patients aged 18 years or older who underwent surgical treatment for spinal metastases (including the entire spinal column from the occipital bone to the sacrum) between January 1^st^, 2018, and December 31^st^, 2022, at two affiliated academic hospitals in Boston: Massachusetts General Hospital (MGH) and Brigham and Women’s Hospital (BWH); or in Utrecht at the University Medical Center Utrecht (UMCU). Patients with spinal localizations of hematological tumors, specifically spinal lymphoma, plasmacytoma and multiple myeloma, were also included due to similarities in surgical treatment [17], [18]. We excluded patients who were treated solely by kyphoplasty or vertebroplasty, radiosurgery, or undergoing revision procedures. The inclusion and exclusion criteria were applied uniformly across both cohorts. Only the first surgical procedure was considered for patients who underwent multiple procedures to maintain statistical independence [19]. The choice of treatment was determined through mutual agreement between both the surgeon and the patient. Fig. 1 illustrates the patient selection process.

### 2.3 Data collection

For the Boston cohort, electronic medical records were manually reviewed to retrospectively retrieve demographic, clinical, oncologic, and operative data. In the Utrecht cohort, data were partially obtained from the three prospective registries and further supplemented for various variables through manual retrospective review of electronic medical records. The same definitions for all variables were applied consistently across both independently collected cohorts.

### 2.4 Demographic, clinical, and oncologic variables

Demographic variables included age, sex, body mass index (BMI), the modified Charlson comorbidity index, and additional comorbidities. Body mass index (BMI) was categorized as underweight (BMI<18.5 kg/m^2^), normal weight (BMI 18.5 to 30 kg/m^2^), and obese (BMI>30 kg/m^2^). The modified Charlson comorbidity index was used to determine comorbidity status [20]. We dichotomized the comorbidity status into any additional comorbidity (in addition to the metastases) or none [21]. Clinical variables included preoperative Eastern Cooperative Oncology Group (ECOG) performance status, the American Society of Anaesthesiologists (ASA) score, preoperative neurologic status using the American Spinal Injury Association (ASIA) impairment scale, Spinal Instability Neoplastic Score (SINS), and Epidural Spinal Cord Compression (ESCC) score. The ECOG performance status was dichotomized into a relatively functional score (0, 1, or 2) or a poor score (3 to 4) [22]. The ASIA impairment scale was used to determine whether a patient had any neurological deficits (score of A, B, C, or D) or none (score of E) [23]. Patients with prior but no current deficits before surgery were classified as ASIA E. The SINS was categorized into stable (0 to 6), potentially unstable (7 to 12), and unstable (13 to 18) [24]. The ESCC score was dichotomized into low-grade ESCC (0 and 1) and high-grade ESCC (2 and 3) [25]. Oncologic variables included primary tumor type, the number of spinal metastases at time of surgery (categorized as one, two, or three or more), region of spinal metastases, the presence of visceral metastases (liver and/or lung); brain metastases or additional bone metastases (outside of the spine), previous systemic therapy, and prior local radiotherapy. Previous systemic therapy was defined as any nonsurgical and non-radiotherapeutic adjuvant treatment (including chemotherapy, immunotherapy, hormone therapy, and metabolic therapy) administered before the index surgery, regardless of dose and response. Prior local radiotherapy was defined as any radiation therapy targeting the operated spinal lesion administered prior to the index surgery, regardless of dose and response.

### 2.5 Surgical management and outcomes

Surgical management aspects included type of surgical procedure, surgical approach, number of spine levels included in the surgery (categorized as one, two, or three or more levels), number of instrumented spine levels (categorized as no, one to three, four to six, or seven or more levels), administration of postoperative local radiotherapy (defined as any form of radiotherapy administered to the spine levels included in the surgery within 3 months after surgery), and the time interval between surgery and start of postoperative local radiotherapy (days). Outcomes included estimated blood loss (milliliters), operating time (minutes), intraoperative complications (including dural tear, nerve injury, and intraoperative death), duration of hospitalization (days), postoperative neurological outcome, systemic postoperative complications within 30 days after surgery, reoperation at surgical site, and 90-day and 1-year survival. Type of surgical procedure was categorized in the following four categories: (1) vertebrectomy/corpectomy with stabilization, (2) open stabilization with or without decompression, (3) percutaneous stabilization with or without minimally invasive decompression, and (4) decompression only. These categories are mutually exclusive to ensure that each patient belonged to no more than one category. Surgical approach was categorized as (1) anterior (ventral), (2) posterior (dorsal), or (3) combined (both anterior and posterior). Estimated blood loss was based on surgeon and anesthesia reports. The duration of hospitalization was defined as the overall number of days patients remained hospitalized during the period when surgery was performed. Postoperative neurological outcome was quantified using the American Spinal Injury Association (ASIA) impairment scale and categorized as either favorable (improved neurological deficit or fully intact neurological status) or unfavorable (unchanged neurological deficit or neurological decline) [26]. Systemic postoperative complications within 30 days included pneumonia, pulmonary embolism, sepsis, myocardial infarction, wound infection, and hardware failure [27]. Reoperation at surgical site was defined as any successive surgical procedure performed during follow-up, without a specific time constraint being recorded. Survival was defined as the time from the surgical procedure until death from any cause, with the last date of follow-up on December 31^st^, 2023 to ensure at least 1-year follow-up. The percentage of patients lost to follow-up was 6% (33/539) in Boston and 5% (9/188) in Utrecht.

### 2.6 Statistical analysis

Categorical variables were described as frequencies with percentages (%), and continuous variables as medians with interquartile ranges (IQR) as histograms suggested non-normal distributions. The Fisher's exact test was used for categorical variables, and the Mann–Whitney *U* test was used for continuous variables, as all continuous variables followed a non-normal distribution. Kaplan-Meier plots illustrated survival curves of both groups. Missing data were handled by complete-case analysis. Two tailed p-values of < 0.05 were considered statistically significant. Statistical analyses were performed using RStudio version 4.3.1 (2023–06-16 UCRT) and Mendeley Reference Manager (2.105.0) was used as reference software.

## 3 Results

### 3.1 Patient populations

**Fig. 2:**
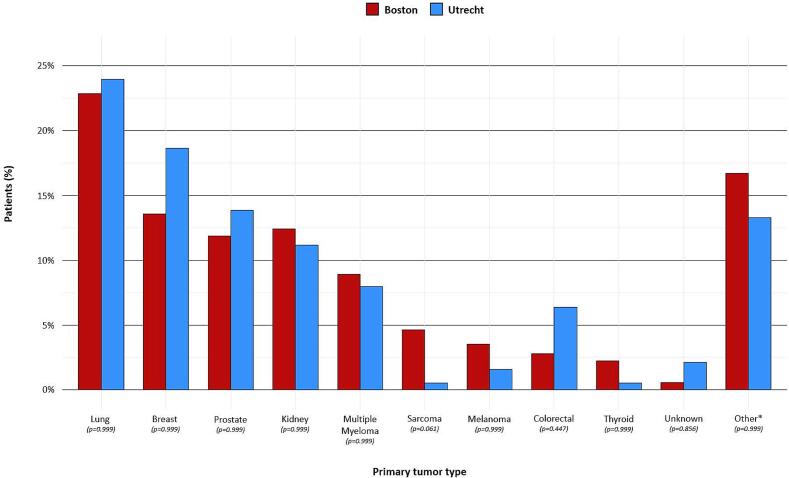
Distribution and comparison of primary tumor types in patients who underwent surgery for spinal metastases in Boston (n=539) and Utrecht (n=188). Fisher’s exact test for count data with Bonferroni correction for multiple testing. *The following cancer types were included in this category for respectively the Boston and Utrecht cohorts: urological cancer (n=15; 2.8%) and (n=5; 2.7%); hepatocellular carcinoma (n=14; 2.6%) and (n=0; 0%); head and neck cancer (n=12; 2.2%) and (n=1; 0.5%); pancreas cancer (n=10; 1.9%) and (n=1; 0.5%); gynaecological cancer (n=8; 1.5%) and (n=5; 2.7%); gallbladder cancer (n=8; 1.5%) and (n=1; 0.5%); malignant lymphoma (n=7; 1.3%) and (n=3; 1.6%); esophageal cancer (n=6; 1.1%) and (n=5; 2.7%); gastric cancer (n=3; 0.6%) and (n=1; 0.5%); squamous cell carcinoma (n=2; 0.4%) and (n=2; 1.1%); pheochromocytoma (n=1; 0.2%) and (n=1; 0.5%); oral cancer (n=1; 0.2%) and (n=0; 0%); pituitary tumor (n=1; 0.2%) and (n=0; 0%); paraganglioma (n=1; 0.2%) and (n=0; 0%); and malignant neoplasm of thymus (n=1; 0.2%) and (n=0; 0%).

In total, 727 patients were included; 539 (74%) patients in Boston and 188 (26%) in Utrecht. In the Boston cohort, a higher percentage of patients had obesity (25% *vs* 16%; p=0.023) and an ASA score of 3 (83% *vs* 47%; p<0.001). In the Utrecht cohort, more patients had a preoperative neurological deficit according to the ASIA impairment scale (47% *vs* 35%; p=0.005). The presence of three or more spinal metastases at the time of surgery was higher in Boston (68% *vs* 59%; p=0.006), with the number of patients with brain metastases also being higher within the Boston cohort (16% *vs* 4.3%; p<0.001). Additionally, more patients received preoperative systemic therapy in Boston than in Utrecht (60% *vs* 44%; p<0.001; Table 1). In both cohorts, the most common primary tumor type was lung cancer, and the second most common primary tumor was breast cancer. Within the Boston cohort, there were more patients with a primary tumor type of sarcoma (4.6% *vs* 0.5%; p=0.061) and melanoma (3.5% *vs* 1.6%; p=0.999), and fewer patients with colorectal tumors (2.8% *vs* 6.4%; p=0.447); however, no differences were found in primary tumor types between both cohorts (all p>0.05; Fig. 2**;** Appendix B).

**Table 1:** Comparison of demographic, clinical, and oncologic characteristics of patients who underwent surgery for spinal metastases in Boston (n=539) or Utrecht (n=188).

**Characteristics**	**N**	**Boston** (*n*=539)	**Utrecht** (*n*=188)	**P-value**
*Demographic*				
Age in years	727	65 (57, 71)	65 (57, 72)	0,509
Female sex	727	239 (44)	81 (43)	0.798
BMI (kg/m^2^)	723†			**0.023**
>30		134 (25)	30 (16)	
18.5–30		384 (71)	150 (82)	
<18.5		21 (3.9)	4 (2.2)	
Modified Charlson comorbidity index	727	8 (7, 10)	9 (8, 9)	0.552
Additional comorbidities§	727	249 (46)	77 (41)	0.233
*Clinical*				
ECOG performance status	664†			>0.999
Score 0 to 2 (≤50% of waking hours bed or chair bound)		403 (84)	155 (84)	
Score 3 to 4 (>50% of waking hours bed or chair bound)		77 (16)	29 (16)	
ASA score	719†			**<0.001**
ASA-1		1 (0.2)	4 (2.2)	
ASA-2		43 (8.0)	77 (43)	
ASA-3		447 (83)	84 (47)	
ASA-4		48 (8.9)	15 (8.3)	
ASIA impairment scale (preoperative)	712†			**0.005**
No neurological deficit (E)		343 (65)	100 (53)	
Neurological deficit (A, B, C, or D)		182 (35)	87 (47)	
SINS category	677†			0.350
Stable		49 (9.9)	16 (8.8)	
Potentially unstable		349 (70)	120 (66)	
Unstable		98 (20)	45 (25)	
ESCC score	677†			0.537
Low-grade ESCC (0 and 1)		97 (19)	35 (21)	
High-grade ESCC (2 and 3)		414 (81)	131 (79)	
*Oncologic*				
Number of spinal metastases at time of surgery	727			**0.006**
1 level		110 (20)	37 (20)	
2 levels		63 (12)	40 (21)	
≥3 levels		366 (68)	111 (59)	
Region of spinal metastases	727			0.077
Cervical		34 (6.3)	4 (2.1)	
Thoracic		168 (31)	55 (29)	
Lumbar		65 (12)	20 (11)	
Combined		272 (50)	109 (58)	
Visceral metastases at time of surgery				
Lung and/or liver	727	242 (45)	88 (47)	0.671
Brain	727	87 (16)	8 (4.3)	**<0.001**
Additional bone metastases at time of surgery	727	354 (66)	113 (60)	0.185
Previous systemic therapy	727	326 (60)	83 (44)	**<0.001**
Prior local radiotherapy to spine levels undergoing surgery	727	137 (25)	37 (20)	0.136

Categorical values were provided as number of patients, with the percentage in parentheses (%) and continuous values as the median and the interquartile range (IQR). Fisher’s exact test and Wilcoxon rank sum test were used to compare both groups. **Bold** p-values indicate statistical significance of p<0.05.

BMI = Body Mass Index; ECOG = Eastern Cooperative Oncology Group performance status; ASA = American Society of Anesthesiologists physical status classification system; ASIA = American Spinal Injury Association impairment scale; SINS = Spinal Instability Neoplastic Score category; ESCC; Epidural Spinal Cord Compression scale; N = number.

† Patient data were respectively available for the Boston cohort and Utrecht cohort, as follows: BMI, 539 (100%) and 184 (98%); ECOG performance status, 480 (89%) and 184 (98%); ASA score, 539 (100%) and 180 (96%); ASIA impairment scale (preoperative), 525 (97%) and 187 (99%); SINS category, 496 (92%) and 181 (96%); ESCC score, 511 (95%) and 166 (88%).

§ These values were based on any comorbidity (addition to the metastases) or none following the modified Charlson comorbidity index.

### 3.2 Surgical management

Vertebrectomy/corpectomy with stabilization was performed in 54% of patients in Boston, whereas none of the patients in Utrecht underwent this procedure (p<0.001). Open stabilization, with or without decompression, was performed in 41% of patients in Boston, compared with 54% of patients in Utrecht (p<0.001). Percutaneous stabilization, with or without minimally invasive decompression, was performed in 1.3% of patients in Boston, compared with 39% of patients in Utrecht (p<0.001). Decompression only was performed in 3.9% of patients in Boston, compared with 6.4% of patients in Utrecht (p<0.001). Additionally, surgeons in Boston used an anterior or combined anterior and posterior approach in 6.6% of cases, whereas surgeons in Utrecht used a posterior approach in all cases (p<0.001). Furthermore, surgeons in Boston operated on three or more spine levels more frequently than surgeons in Utrecht (97% *vs* 94%; p=0.004), and they also more frequently instrumented seven or more spine levels (27% *vs* 3.2%; p<0.001). Postoperative local radiotherapy was administered in 54% of patients in Boston and 70% of patients in Utrecht (p<0.001). Additionally, postoperative local radiotherapy was initiated a median of 12 days earlier in patients in Utrecht compared with patients in Boston (17 days [IQR 12–24] *vs* 29 days [IQR 23–39]; p<0.001; Table 2).

**Table 2:** Comparison of surgical management between Boston (n=539) and Utrecht (n=188).

**Characteristics**	**N**	**Boston** (*n*=539)	**Utrecht** (*n*=188)	**P-value**
Type of surgical procedure	727			**<0.001**
Vertebrectomy/corpectomy with stabilization		289 (54)	0 (0)	
Open stabilization with or without decompression		222 (41)	102 (54)	
Percutaneous stabilization with or without minimally invasive decompression		7 (1.3)	74 (39)	
Decompression only		21 (3.9)	12 (6.4)	
Surgical approach	727			**<0.001**
Anterior (ventral)		25 (4.6)	0 (0)	
Posterior (dorsal)		503 (93)	188 (100)	
Combined (both posterior and anterior)		11 (2.0)	0 (0)	
Number of spine levels included in the surgery	727			**0.004**
1 level		0 (0)	4 (2.1)	
2 levels		16 (3.0)	8 (4.3)	
≥3 levels		523 (97)	176 (94)	
Number of instrumented spine levels	727			**<0.001**
No levels		21 (3.9)	12 (6.4)	
1 to 3 levels		110 (20)	76 (40)	
4 to 6 levels		262 (49)	94 (50)	
≥7 levels		146 (27)	6 (3.2)	
Postoperative local radiotherapy	727	290 (54)	132 (70)	**<0.001**
Interval from surgery to start postoperative local radiotherapy (days)	417†	29 (23, 39)	17 (12, 24)	**<0.001**

Categorical values were provided as number of patients, with the percentage in parentheses (%) and continuous values as the median and the interquartile range (IQR). Fisher’s exact test and Wilcoxon rank sum test were used to compare both groups. **Bold** p-values indicate statistical significance of p<0.05.

† Patient data were respectively available for the Boston cohort and Utrecht cohort, as follows: interval from surgery to start postoperative local radiotherapy (days), 287/290 (99%) and 130/132 (98%).

### 3.3 Perioperative outcomes and survival

Patients in Boston compared with patients in Utrecht experienced approximately twice as much blood loss during surgery (500 ml [IQR 250–1000] *vs* 250 ml [IQR 150–500]; p<0.001) and had nearly twice the duration of surgery (240 min [IQR 178–305] *vs* 131 min [IQR 99–164]; p<0.001). The occurrence of intraoperative complications did not differ between both cohorts (5.8% *vs* 2.7%; p=0.117). The median total duration of hospitalization was two days longer for patients in Boston compared with those in Utrecht (8 days [IQR 5–12] *vs* 6 days [IQR 4–12]; p=0.017). Favorable postoperative neurological outcome (78% *vs* 76%; p=0.477), occurrence of 30-day systemic postoperative complications (15% *vs* 15%; p=0.906), and reoperations at surgical site (14% *vs* 14%; p>0.999) did not differ between both cohorts. The 90-day (79% *vs* 78%; p=0.792) and 1-year survival rates (49% *vs* 50%; p=0.882; Appendix C) also did not differ (Table 3).

**Table 3:** Comparison of perioperative outcomes and survival between Boston (n=539) and Utrecht (n=188).

**Outcomes**	**N**	**Boston** (*n*=539)	**Utrecht** (*n*=188)	**P-value**
Estimated blood loss (ml)	686†	500 (250, 1,000)	250 (150, 500)	**<0.001**
Operating time (min)	727	240 (178, 305)	131 (99, 164)	**<0.001**
Intraoperative complications	727	31 (5.8)	5 (2.7)	0.117
Duration of hospitalization (days)	727	8 (5, 12)	6 (4, 12)	**0.017**
Postoperative neurological outcome	722†			0.477
Favorable		418 (78)	142 (76)	
Unfavorable		116 (22)	46 (24)	
Systemic postoperative complications within 30 days after surgery	727	80 (15)	29 (15)	0.906
Reoperations	727	75 (14)	26 (14)	>0.999
Survival				
90-day	719†	424 (79)	145 (78)	0.792
1-year	685†	247 (49)	89 (50)	0.882

Categorical values were provided as number of patients, with the percentage in parentheses (%) and continuous values as the median and the interquartile range (IQR). Fisher’s exact test and Wilcoxon rank sum test were used to compare both groups. **Bold** p-values indicate statistical significance of p<0.05.

ml = milliliters; min = minutes.

† Patient data were respectively available for the Boston cohort and Utrecht cohort, as follows: estimated blood loss (ml), 531 (99%) and 155 (82%); postoperative neurological outcome, 534 (99%) and 188 (100%); 90-day survival, 534 (99%) and 185 (98%); 1-year survival, 506 (94%) and 179 (95%).

## 4 Discussion

Currently, there is no international consensus on the surgical management or standardized treatment planning for patients with symptomatic spinal metastases requiring surgical intervention. Knowledge of variations in surgical care may offer valuable insights to guide surgical decision-making and support research efforts toward improving patient care. In our cross-continental cohort study, we identified differences in patient populations and surgical management between major cancer centers in Boston and Utrecht. Patients in Boston generally presented with a more advanced stage of metastatic disease at the time of surgery compared with patients in Utrecht. Notably, surgeons in Utrecht favoured less invasive, posterior techniques, while surgeons in Boston performed more extensive surgeries, including procedures such as corpectomy/vertebrectomy. Additionally, patients in Utrecht experienced less intraoperative blood loss, shorter operating times, and shorter hospital stays compared with patients in Boston; however, no differences in postoperative neurological outcome, complications, reoperation rates or survival were observed.

### 4.1 Patient populations

Overall, the Boston and Utrecht cohorts were largely comparable in demographic characteristics, including age, sex, and the presence of comorbidities. Additionally, the distributions of ECOG performance status, SINS category, and ESCC score were similar between both cohorts. Patients in the Boston cohort generally had a more advanced stage of metastatic disease at the time of surgery, as evidenced by a higher number of spinal metastases and more instances of brain metastases. One potential explanation for this difference in the stage of metastatic disease could be attributed to Utrecht’s recent efforts, prior to the start of inclusion in 2018, to identify patients with symptomatic spinal metastases earlier to prevent delays in treatment [28], [29]. Furthermore, the more advanced stage of metastatic disease in the Boston cohort may explain the higher rate of preoperative systemic therapy. Since a more advanced stage of disease is associated with a poorer prognosis, assessing the response to systemic therapy before considering surgery may have been preferred in certain cases in Boston [30]. However, differences in clinical protocols and treatment guidelines between the United States and the Netherlands, particularly regarding the timing of systemic therapy, may also have contributed to the observed variation. The difference in the stage of metastatic disease, along with the higher proportion of patients with obese (BMI>30 kg/m^2^) in the Boston cohort, could serve as an explanation for the difference in ASA score between both cohorts. However, differences in ASA scoring are likely to be further influenced by local regulations on availability of surgery or “upcoding”, where clinicians may have had incentives to underestimate or overestimate the ASA class to support their service, resulting in variations between countries [31]. The observed differences in patient populations between Boston and Utrecht, specifically the higher number of spinal metastases, may partly explain the variations in surgical management, as a more advanced stage of metastatic spinal disease can drive the decision to pursue more aggressive surgical approaches. Conversely, earlier identification and management of patients at an earlier stage of metastatic disease may allow for less invasive surgical approaches.

### 4.2 Variation in surgical management

In general, more extensive surgical procedures were performed in Boston compared with Utrecht. Specifically, open vertebrectomies/corpectomies involving substantial tumor resection were commonly used in Boston, whereas percutaneous surgery was predominantly used in Utrecht. Additionally, a greater number of patients in Utrecht received postoperative radiotherapy, with radiation starting a median of 12 days earlier than in Boston. The observed differences in surgery and postoperative radiotherapy may be attributed to differing treatment philosophies across centers. In Utrecht, the extensive use of percutaneous surgery and the higher number of patients receiving postoperative radiotherapy result from an increased collaboration between radiation oncologists and spine surgeons, which started in 2015 and has facilitated a clear allocation of responsibilities and tasks. This intensified collaboration means that if local tumor control can effectively be achieved through radiotherapy, spine surgeons primarily focus on restoring or maintaining spinal stability, minimizing mechanical (activity-related) pain, and facilitating the safe delivery of radiotherapy. Consequently, over the past decade, Utrecht has shifted towards using postoperative radiotherapy rather than surgical resection as the primary strategy to achieve local tumor control [28], [29]. Alternatively in Boston, contemporary surgical procedures involving substantial tumor resection for cytoreduction are still regularly performed, suggesting a different strategy in which surgical resection continues to play a central role to achieve local tumor control. Differences in surgical strategies between North American and European centers have been previously noted, with Wright et al. [31] reporting that procedures involving resection of over 50 % of the metastatic lesion were more common in North American centers, whereas European centers more frequently performed procedures involving resection of less than 50 % of the metastatic lesion. Such variations in surgical strategies and treatment philosophies are likely influenced by a combination of financial, institutional, and cultural factors. An example of this is the relatively lower reimbursement for percutaneous procedures in the United States compared to more extensive surgeries [32]. Furthermore, cultural differences between the United States and the Netherlands regarding end-of-life care may also influence the difference in surgical management [33]. In the United States, clinicians tend to pursue more aggressive and resource-intensive treatment in end-of-life care, as evidenced by the higher rates of chemotherapy utilization and ICU admission among patients dying with cancer [34]. In the Netherlands, by contrast, end-of-life care is largely focused on conforming to patient preferences and symptom management [35], [36]. Finally, surgeons' preferences and training are also likely to have played a significant role in this observed variation in surgical management.

### 4.3 Perioperative outcomes and survival

Patients in Utrecht experienced lower rates of intraoperative blood loss, shorter operating times and shorter total hospital stays compared with patients in Boston, possibly due to the adoption of less invasive surgical techniques. Pennington et al. [37] reported similar results when comparing minimally invasive versus conventional spine surgery for spinal metastases. Notably, the higher number of instrumented spine levels in the Boston cohort may have contributed to the observed differences in intraoperative blood loss and operating times. Despite variations in surgical management strategies, no differences were found between Boston and Utrecht in terms of intraoperative complications, postoperative neurological outcome, 30-day systemic postoperative complications, reoperations, or 90-day and 1-year survival. These findings are partially in line with the literature, as Hansen-Algenstaedt et al. [38] and Miscusi et al. [39] both reported similar findings, stating no difference in intraoperative and systemic postoperative complications in patients with spinal metastases treated with less invasive surgeries. Furthermore, although patients in Utrecht presented with a higher prevalence of preoperative neurological deficit and underwent less extensive decompressive surgery, postoperative neurological outcome was similar between the two cohorts, suggesting that minimally invasive decompression may be as effective as more extensive decompressive procedures, such as vertebrectomy/corpectomy. The similarities in postoperative neurological outcome, complication rates, and survival suggest that more extensive surgical procedures, which seem to be associated with increased intraoperative blood loss, longer surgeries and longer hospital stays, did not necessarily result in better clinical outcomes. However, these findings may have been influenced by the worse prognosis of Boston patients, who presented with more advanced metastatic disease at the time of surgery.

### 4.4 Implications for clinical practice

This study demonstrates that while there were differences in patient populations and surgical management across major cancer centers in two distinct countries, the clinical outcomes remained largely comparable. These findings suggest that if patients with symptomatic spinal metastases undergo surgery at an earlier stage of metastatic disease, percutaneous stabilization procedures combined with radiotherapy for local tumor control and pain alleviation may achieve similar survival outcomes when compared to more contemporary extensive surgical approaches. However, given the limited survival of patients with spinal metastases, treatment is increasingly focused on improving or preserving quality of life. It is conceivable that surgical management strategies involving less invasive procedures may improve quality of life more effectively, given the higher morbidity associated with more aggressive surgical approaches. Therefore, clinicians should carefully consider the extent of surgery required for patients with symptomatic spinal metastases and make efforts to prevent treatment delays to allow for minimally invasive surgical strategies at an earlier stage of metastatic disease. Future research should focus on evaluating integrated strategies that combine less invasive surgery with radiotherapy, as well as the extent and timing of surgery needed to improve or preserve quality of life for patients with spinal metastases.

### 4.5 Limitations

This study had several limitations. First, we were unable to determine the number of patients treated non-surgically among all patients treated for spinal metastases across different institutions. Consequently, we could not examine potential differences in patient selection for surgery; instead, we could only address differences in patients selected fit for surgery. Second, we were unable to investigate a definitive cause for the variation in surgical management. This likely stems from multiple factors, including cultural differences regarding end-of-life care, surgeons' training, increased focus on multidisciplinary collaboration between radiation oncologists and spine surgeons, and possible financial incentives, such as reimbursements [32]. Ideally, we would have collected data related to surgical treatment decision-making, but were unable to due to the retrospective design. Third, we were unable to compare patient satisfaction and quality-of-life outcomes, as these were not documented during routine case visits in Boston. Including such information could have further enhanced the impact of this study. Additionally, given the rising incidence of spinal metastases, it would have been interesting to study the cost-effectiveness of the varying surgical management across institutions, as this could provide insights into the financial implications of different treatment strategies. Furthermore, it would have been valuable to categorize separation surgery as a distinct type of surgical procedure. However, due to its relatively recent development and ongoing evolution, the technique remains insufficiently defined, resulting in substantial variability in its application across institutions, which precluded a meaningful comparison [40], [41]. Finally, SINS scores were not always reported and had to be manually extracted from patient records. In Boston, pain characteristics such as mechanical pain were rarely included in admission notes or radiology reviews. However, we believe we were able to extract pain reasonably accurately from the consultation reference notes in the medical records. Despite these limitations, our large cross-continental cohort study enabled us to provide valuable insights into the differences in patients selected for surgery, surgical management, and outcomes between cross-continental tertiary institutions. Future studies investigating different surgical management strategies should include prospectively collected quality-of-life outcomes, patient satisfaction, and factors influencing treatment decision-making.

## 5 Conclusion

This retrospective study of patients surgically treated for spinal metastases identified differences in patient populations and surgical management between major cancer centers on separate continents. Overall, patients undergoing surgery for spinal metastases in Boston generally presented with a more advanced stage of metastatic disease at the time of surgery compared with patients in Utrecht. Additionally, the use of more extensive surgical procedures, such as anterior column resection, was greater in Boston, whereas in Utrecht, patients received postoperative radiotherapy more frequently and earlier than in Boston. Further research is needed to evaluate surgical management strategies that integrate less invasive surgery with radiotherapy and determine the required extent and optimal timing of surgery to improve the quality of life for patients with spinal metastases.

## 6 Institutions

Investigation was performed at the Department of Orthopaedic Surgery, Massachusetts General Hospital - Harvard Medical School, Boston, United States and the Department of Orthopaedic Surgery, University Medical Center Utrecht, the Netherlands.

## 7 IRB approval

2017P00838 (MGH) and VOBU 23U-0528 (UMCU).

## 8 Data availability statement

The datasets generated during and/or analysed during the current study are not publicly available due to legal restrictions arising from data-sharing agreements but are available from the corresponding author on reasonable request.

## CRediT authorship contribution statement

**Jantijn J.G.J. Amelink:** Writing – review & editing, Writing – original draft, Methodology, Formal analysis, Data curation, Conceptualization. **Bram T. van Munster:** Writing – review & editing, Writing – original draft, Data curation, Conceptualization. **Bas J.J. Bindels:** Writing – review & editing, Validation, Methodology, Data curation. **Robertus J.B. Pierik:** Writing – review & editing, Project administration, Data curation. **Jasper van Tiel:** Writing – review & editing, Validation. **Olivier Q. Groot:** Writing – review & editing, Validation, Supervision, Methodology, Conceptualization. **Nicolien Kasperts:** Writing – review & editing, Validation. **Daniel G. Tobert:** Writing – review & editing, Validation, Supervision, Conceptualization. **Jorrit-Jan Verlaan:** Writing – review & editing, Validation, Supervision, Conceptualization.

## Declaration of competing interest

The authors declare that they have no known competing financial interests or personal relationships that could have appeared to influence the work reported in this paper.
