# Supplementary material for: Surgical management of spinal metastases: A cross-continental study in the United States and the Netherlands
Source: J Bone Oncol. 2025 Mar 25;52:100676. doi: 10.1016/j.jbo.2025.100676 (PMC11994354; doi:10.1016/j.jbo.2025.100676)
Supplement: Supplementary Data 2 [file mmc2.docx]

**Appendix B. Origin of primary tumors of patients who underwent surgery for spinal metastases in Boston (n=539) or Utrecht (n=188)**

| **Characteristics** | **Boston**  (*n*=539) | **Utrecht**  (*n*=188) | **P-value**) |
| --- | --- | --- | --- |
| Type of primary tumor, n (%) |  |  |  |
| Lung cancer | 123 (23%) | 45 (24%) | 0.999 |
| Breast cancer | 73 (14%) | 35 (19%) | 0.999 |
| Prostate cancer | 64 (12%) | 26 (14%) | 0.999 |
| Kidney cancer | 67 (12%) | 21 (11%) | 0.999 |
| Multiple myeloma | 48 (8.9%) | 15 (8.0%) | 0.999 |
| Sarcoma | 25 (4.6%) | 1 (0.5%) | 0.061 |
| Melanoma | 19 (3.5%) | 3 (1.6%) | 0.999 |
| Colorectal cancer | 15 (2.8%) | 12 (6.4%) | 0.447 |
| Thyroid cancer | 12 (2.2%) | 1 (0.5%) | 0.999 |
| Unknown | 3 (0.6%) | 4 (2.1%) | 0.856 |
| Other* | 90 (17%) | 25 (13%) | 0.999 |
| Fisher’s exact test for count data with Bonferroni correction for multiple testing.  *The following cancer types were included in this category for respectively the Boston and Utrecht cohorts: urological cancer (n=15; 2.8%) and (n=5; 2.7%); hepatocellular carcinoma (n=14; 2.6%) and (n=0; 0%); head and neck cancer (n=12; 2.2%) and (n=1; 0.5%); pancreas cancer (n=10; 1.9%) and (n=1; 0.5%); gynaecological cancer (n=8; 1.5%) and (n=5; 2.7%); gallbladder cancer (n=8; 1.5%) and (n=1; 0.5%); malignant lymphoma (n=7; 1.3%) and (n=3; 1.6%); esophageal cancer (n=6; 1.1%) and (n=5; 2.7%); gastric cancer (n=3; 0.6%) and (n=1; 0.5%); squamous cell carcinoma (n=2; 0.4%) and (n=2; 1.1%); pheochromocytoma (n=1; 0.2%) and (n=1; 0.5%); oral cancer (n=1; 0.2%) and (n=0; 0%); pituitary tumor (n=1; 0.2%) and (n=0; 0%); paraganglioma (n=1; 0.2%) and (n=0; 0%); and malignant neoplasm of thymus (n=1; 0.2%) and (n=0; 0%). | | | |
