# Supplementary material for: Surgical management of spinal metastases: A cross-continental study in the United States and the Netherlands
Source: J Bone Oncol. 2025 Mar 25;52:100676. doi: 10.1016/j.jbo.2025.100676 (PMC11994354; doi:10.1016/j.jbo.2025.100676)
Supplement: Supplementary Data 3 [file mmc3.docx]

**Appendix C. Kaplan-Meier curves for 1-year survival**

**
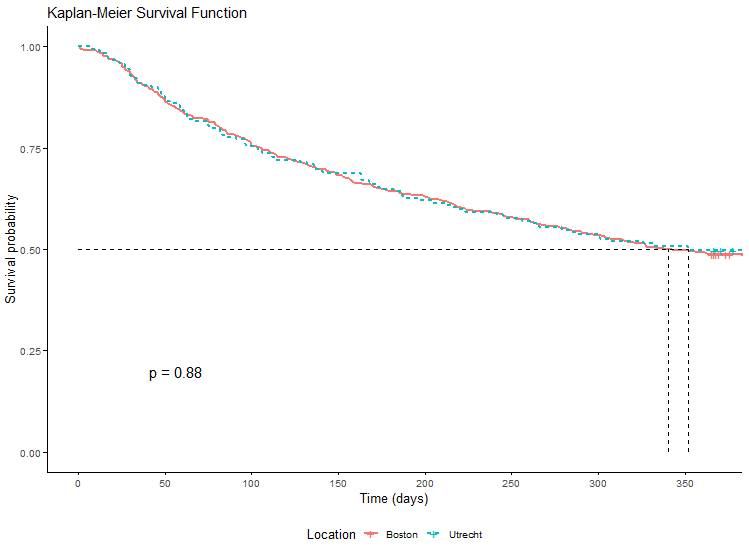
**

Kaplan-Meier survival curves for patients operated in Boston and Utrecht for spinal metastases
